# 7,7′,8,8′-Tetra­meth­oxy-4,4′-dimethyl-3,5′-bichromene-2,2′-dione

**DOI:** 10.1107/S1600536809018029

**Published:** 2009-05-20

**Authors:** Hoong-Kun Fun, Samuel Robinson Jebas, Mehtab Parveen, Zakia Khanam, Raza Murad Ghalib

**Affiliations:** a X-ray Crystallography Unit, School of Physics, Universiti Sains Malaysia, 11800 Universiti Sains Malaysia, Penang, Malaysia; b Department of Chemistry, Aligarh Muslim University, Aligarh 202 002 (UP), India

## Abstract

In the title mol­ecule, C_24_H_22_O_8_, the mean planes of the two coumarin units are inclined to each other at a dihedral angle of 79.93 (3)°. The attached meth­oxy groups form torsion angles of 7.65 (19) and 78.36 (14)° with respect to one coumarin unit, and angles of 9.01 (16) and 99.08 (11)° with respect to the other coumarin unit. In the crystal structure, weak inter­molecular C—H⋯O hydrogen bonds connect pairs of mol­ecules to form dimers, generating *R*_2_^2^(16) and *R*_2_^2^(18) rings; the dimers are linked by further weak inter­molecular C—H⋯O hydrogen bonds, forming extended chains. Additional stabil­ization is provided by weak C—H⋯π inter­actions.

## Related literature

For the biological activity of coumarins, see: El-Agrody *et al.* (2001 ▶); El-Farargy (1991 ▶); Emmanuel-Giota *et al.* (2001 ▶); Ghate *et al.* (2005 ▶); Laakso *et al.* (1994 ▶); Nofal *et al.* (2000 ▶); Pratibha *et al.* (1999 ▶); Shaker (1996 ▶); Yang *et al.* (2005 ▶). For the pharmaceutical properties of coumarin derivatives, see: Kennedy *et al.* (1997 ▶). For related literature on natural and synthetic coumarins, see: Carlton *et al.* (1996 ▶); Zhou *et al.* (2000 ▶). For standard bond-length data, see: Allen *et al.* (1987 ▶). For the stability of the temperature controller used in the data collection, see: Cosier & Glazer (1986 ▶). For hydrogen-bond motifs, see: Bernstein *et al.* (1995 ▶).
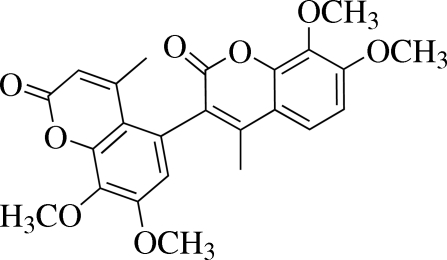


## Experimental

### 

#### Crystal data

C_24_H_22_O_8_
*M* *_r_* = 438.42
Monoclinic, 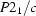

*a* = 9.4724 (1) Å
*b* = 23.4766 (3) Å
*c* = 9.3525 (1) Å
β = 96.254 (1)°
*V* = 2067.43 (4) Å^3^
*Z* = 4
Mo *K*α radiation
μ = 0.11 mm^−1^
*T* = 100 K
0.50 × 0.27 × 0.14 mm

#### Data collection

Bruker SMART APEXII CCD area-detector diffractometer
Absorption correction: multi-scan (*SADABS*; Bruker, 2005 ▶) *T*_min_ = 0.949, *T*_max_ = 0.985
58385 measured reflections
7006 independent reflections
6023 reflections with *I* > 2σ(*I*)
*R*_int_ = 0.030

#### Refinement

*R*[*F* ^2^ > 2σ(*F* ^2^)] = 0.046
*wR*(*F* ^2^) = 0.125
*S* = 1.07
7006 reflections
295 parameters
H-atom parameters constrained
Δρ_max_ = 0.47 e Å^−3^
Δρ_min_ = −0.24 e Å^−3^

### 

Data collection: *APEX2* (Bruker, 2005 ▶); cell refinement: *SAINT* (Bruker, 2005 ▶); data reduction: *SAINT*; program(s) used to solve structure: *SHELXTL* (Sheldrick, 2008 ▶); program(s) used to refine structure: *SHELXTL*; molecular graphics: *SHELXTL*; software used to prepare material for publication: *SHELXTL* and *PLATON* (Spek, 2009 ▶).

## Supplementary Material

Crystal structure: contains datablocks global, I. DOI: 10.1107/S1600536809018029/lh2822sup1.cif

Structure factors: contains datablocks I. DOI: 10.1107/S1600536809018029/lh2822Isup2.hkl

Additional supplementary materials:  crystallographic information; 3D view; checkCIF report

## Figures and Tables

**Table 1:** Hydrogen-bond geometry (Å, °)

*D*—H⋯*A*	*D*—H	H⋯*A*	*D*⋯*A*	*D*—H⋯*A*
C21—H21*A*⋯O6^i^	0.96	2.55	3.2921 (15)	134
C22—H22*A*⋯O6^ii^	0.96	2.52	3.4385 (14)	161
C22—H22*B*⋯O8^i^	0.96	2.56	3.4401 (15)	152
C6—H6*A*⋯*Cg*1^iii^	0.93	2.92	3.6706 (12)	138
C19—H19*A*⋯*Cg*2^iv^	0.96	2.60	3.5446 (14)	170

Symmetry codes: (i) 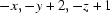; (ii) 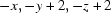; (iii) 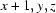; (iv) 
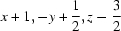
. *Cg*1 is the centroid of the O7/C14–C18 ring and *Cg*2 is the centroid of the C10–C18 ring.

**Fig. 1.**
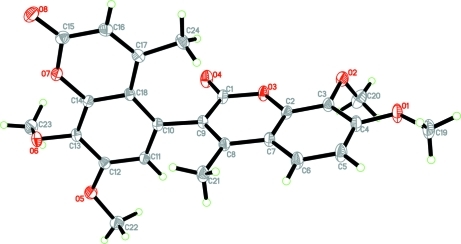
The molecular structure of (I), showing 50% probability displacement ellipsoids and the atom numbering scheme.

**Fig. 2.**
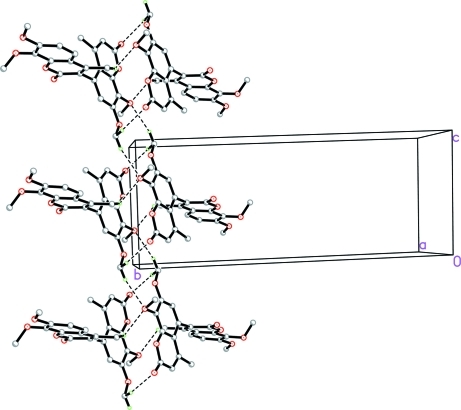
Part of the crystal structure of (I). Dashed lines indicate the hydrogen bonds.

## supplementary crystallographic information

### Comment

Coumarins are a large group of naturally occurring oxygen heterocycles representing 2*H*-1-benzopyran-2-one derivatives. Many natural coumarins are reputed for their wide range of biological activites such as antibacterial (El-Agrody *et al.*, 2001; Pratibha *et al.*, 1999), antifungal (Shaker, 1996; El-Farargy, 1991), antioxidant (Yang *et al.*, 2005), analgesic (Ghate *et al.*, 2005), anti-inflammatory (Emmanuel-Giota *et al.*, 2001) and antitumor (Nofal *et al.*, 2000). Bi and tri-coumarins are a comparatively new group of compounds which are widespread in nature and their biological properties are also well known (Laakso *et al.*, 1994). One of the characteristic pharmacological properties of coumarin derivatives is anticoagulant action (Kennedy *et al.*, 1997). A large number of natural and semisynthetic coumarin and bicoumarin derivatives have been reported to demonstrate chemopreventive (Carlton *et al.*, 1996) and anti-HIV (Zhou *et al.*, 2000) activities. Keeping in view of these biological importance of coumarins and their dimers, we have synthesized the title compound (I) and report herein its crystal structure.

The molecular structure of the title compound is shown in Fig .1. In crystal structure of (I) molecules are linked by weak intermolecular C-H···O hydrogen bonds to form *R*_2_^2^(16) and *R*_2_^2^(18) rings (Bernstein *et al.*, (1995). The two coumarin units are essentially planar with the maximum deviation from planarity of 0.0665 (11)Å for atom C9 in the ring (O3/C1–C9) and 0.0419 (12)Å for atom C16 in the ring (O7/C10–C18). The two coumarin units forming a dihedral angle of 79.93 (3)° (O3/C1–C9:O7/C10–C18), indicating that they are inclined to each other. Two of the methoxy units attached to the each coumarin units are twisted from the plane of coumarin unit as indicated by the torsion angles of C19–O1—C4–C5=-7.65 (19)°; C20–O2–C3–C2=78.36 (14)°; C22–O5–C12–C11=9.01 (16)° and C23–O6–C13–C14=99.08 (11)°, respectively. The bond lengths Allen *et al.* (1987) and bond angles are normal.

The crystal packing is illustrated in Fig. 2. In addition C—H···π interactions help stabilize the crystal structure.

### Experimental

A mixture of 7,8-dimethoxy-4-methyl coumarin (2.20 g, 10 mmol) and manganese(III) acetate (0.774 g, 1 mmol) was stirred at room temperature, then 70% perchloric acid (0.8 g, 6 mmol) was added. The reaction mixture was heated under reflux at 114°C with stirring in the atmosphere of nitrogen for 3 h. The reaction mixture was cooled and diluted with 50 ml of benzene. The benzene solution was washed with water and aq. NaHCO_3_, dried over anhydrous Na_2_SO_4_ and left to evaporate. The residue showed two major compounds which were separated by column chromatography followed by preparative thin layer chromatography (Benzene: EtOAc, 9:1) into the title compound (I) (260 mg, 12%).

### Refinement

H atoms were positioned geometrically [C–H = 0.93–0.96 Å] and refined using a riding model with *U*_iso_(H) = 1.2*U*_eq_(C) and 1.5*U*_eq_(methyl C). A rotating–group model was used for the methyl groups.

### Crystal data

**Table:**

C_24_H_22_O_8_	*F*(000) = 920
*M**_r_* = 438.42	*D*_x_ = 1.409 Mg m^−^^3^
Monoclinic, *P*2_1_/*c*	Mo *K*α radiation, λ = 0.71073 Å
Hall symbol: -P 2ybc	Cell parameters from 9875 reflections
*a* = 9.4724 (1) Å	θ = 2.7–33.0°
*b* = 23.4766 (3) Å	µ = 0.11 mm^−^^1^
*c* = 9.3525 (1) Å	*T* = 100 K
β = 96.254 (1)°	Plate, colourless
*V* = 2067.43 (4) Å^3^	0.50 × 0.27 × 0.14 mm
*Z* = 4	

### Data collection

**Table:**

Bruker SMART APEXII CCD area-detector diffractometer	7006 independent reflections
Radiation source: fine-focus sealed tube	6023 reflections with *I* > 2σ(*I*)
graphite	*R*_int_ = 0.030
φ and ω scans	θ_max_ = 31.7°, θ_min_ = 1.7°
Absorption correction: multi-scan (*SADABS*; Bruker, 2005)	*h* = −13→13
*T*_min_ = 0.949, *T*_max_ = 0.985	*k* = −34→34
58385 measured reflections	*l* = −13→13

### Refinement

**Table:**

Refinement on *F*^2^	Primary atom site location: structure-invariant direct methods
Least-squares matrix: full	Secondary atom site location: difference Fourier map
*R*[*F*^2^ > 2σ(*F*^2^)] = 0.046	Hydrogen site location: inferred from neighbouring sites
*wR*(*F*^2^) = 0.125	H-atom parameters constrained
*S* = 1.07	*w* = 1/[σ^2^(*F*_o_^2^) + (0.0593*P*)^2^ + 0.8142*P*] where *P* = (*F*_o_^2^ + 2*F*_c_^2^)/3
7006 reflections	(Δ/σ)_max_ < 0.001
295 parameters	Δρ_max_ = 0.47 e Å^−^^3^
0 restraints	Δρ_min_ = −0.23 e Å^−^^3^

### Special details

**Table:**

Experimental. The crystal was placed in the cold stream of an Oxford Cyrosystems Cobra open-flow nitrogen cryostat (Cosier & Glazer, 1986) operating at 100.0 (1) K.
Geometry. All e.s.d.'s (except the e.s.d. in the dihedral angle between two l.s. planes) are estimated using the full covariance matrix. The cell e.s.d.'s are taken into account individually in the estimation of e.s.d.'s in distances, angles and torsion angles; correlations between e.s.d.'s in cell parameters are only used when they are defined by crystal symmetry. An approximate (isotropic) treatment of cell e.s.d.'s is used for estimating e.s.d.'s involving l.s. planes.
Refinement. Refinement of *F*^2^ against ALL reflections. The weighted *R*-factor *wR* and goodness of fit *S* are based on *F*^2^, conventional *R*-factors *R* are based on *F*, with *F* set to zero for negative *F*^2^. The threshold expression of *F*^2^ > σ(*F*^2^) is used only for calculating *R*-factors(gt) *etc*. and is not relevant to the choice of reflections for refinement. *R*-factors based on *F*^2^ are statistically about twice as large as those based on *F*, and *R*- factors based on ALL data will be even larger.

### Fractional atomic coordinates and isotropic or equivalent isotropic displacement parameters (Å^2^)

**Table:**

	*x*	*y*	*z*	*U*_iso_*/*U*_eq_	
O1	0.69654 (9)	0.68242 (4)	0.29953 (11)	0.0282 (2)	
O2	0.43643 (8)	0.65723 (3)	0.37372 (10)	0.02296 (17)	
O3	0.26852 (8)	0.73969 (3)	0.46388 (9)	0.01987 (16)	
O4	0.05685 (9)	0.76180 (3)	0.52477 (10)	0.02502 (18)	
O5	0.03012 (9)	0.95276 (4)	0.88144 (9)	0.02254 (17)	
O6	−0.18774 (8)	0.99197 (3)	0.69969 (9)	0.02027 (16)	
O7	−0.22134 (8)	0.96109 (3)	0.42722 (8)	0.01782 (15)	
O8	−0.35251 (9)	0.97343 (4)	0.21952 (10)	0.02664 (18)	
C1	0.17182 (11)	0.77961 (4)	0.50060 (12)	0.01762 (19)	
C2	0.40061 (11)	0.75552 (4)	0.43041 (12)	0.01703 (19)	
C3	0.48594 (11)	0.71220 (4)	0.38541 (13)	0.0191 (2)	
C4	0.62110 (12)	0.72617 (5)	0.34739 (13)	0.0217 (2)	
C5	0.66991 (12)	0.78230 (5)	0.36156 (16)	0.0274 (3)	
H5A	0.7607	0.7914	0.3398	0.033*	
C6	0.58343 (12)	0.82435 (5)	0.40787 (15)	0.0247 (2)	
H6A	0.6174	0.8615	0.4172	0.030*	
C7	0.44578 (11)	0.81239 (4)	0.44116 (12)	0.01799 (19)	
C8	0.34781 (11)	0.85546 (4)	0.48140 (12)	0.01690 (18)	
C9	0.21437 (11)	0.83939 (4)	0.50522 (11)	0.01574 (18)	
C10	0.10535 (11)	0.88049 (4)	0.54652 (11)	0.01548 (18)	
C11	0.12224 (11)	0.89822 (4)	0.68924 (11)	0.01727 (19)	
H11A	0.1996	0.8852	0.7502	0.021*	
C12	0.02494 (11)	0.93527 (4)	0.74272 (11)	0.01696 (18)	
C13	−0.09034 (11)	0.95541 (4)	0.65136 (11)	0.01643 (18)	
C14	−0.10474 (10)	0.93843 (4)	0.50800 (11)	0.01503 (18)	
C15	−0.24804 (11)	0.95111 (5)	0.28163 (12)	0.0192 (2)	
C16	−0.15038 (12)	0.91360 (5)	0.21998 (12)	0.01956 (19)	
H16A	−0.1646	0.9067	0.1215	0.023*	
C17	−0.03892 (11)	0.88778 (4)	0.29753 (11)	0.01678 (18)	
C18	−0.00961 (10)	0.90076 (4)	0.45039 (11)	0.01475 (17)	
C19	0.82791 (13)	0.69680 (6)	0.24429 (17)	0.0318 (3)	
H19A	0.8643	0.6639	0.1997	0.048*	
H19B	0.8956	0.7094	0.3218	0.048*	
H19C	0.8115	0.7267	0.1745	0.048*	
C20	0.49521 (14)	0.62163 (6)	0.48972 (18)	0.0333 (3)	
H20A	0.4725	0.5826	0.4674	0.050*	
H20B	0.4560	0.6322	0.5763	0.050*	
H20C	0.5965	0.6262	0.5031	0.050*	
C21	0.39362 (12)	0.91653 (5)	0.49091 (15)	0.0257 (2)	
H21A	0.3114	0.9406	0.4878	0.039*	
H21B	0.4459	0.9254	0.4115	0.039*	
H21C	0.4528	0.9227	0.5796	0.039*	
C22	0.15479 (13)	0.93845 (5)	0.97486 (12)	0.0238 (2)	
H22A	0.1452	0.9520	1.0701	0.036*	
H22B	0.2362	0.9559	0.9401	0.036*	
H22C	0.1667	0.8978	0.9770	0.036*	
C23	−0.29354 (12)	0.96294 (5)	0.77235 (14)	0.0259 (2)	
H23A	−0.3698	0.9887	0.7853	0.039*	
H23B	−0.2515	0.9498	0.8645	0.039*	
H23C	−0.3297	0.9310	0.7156	0.039*	
C24	0.04626 (13)	0.84603 (5)	0.22071 (12)	0.0231 (2)	
H24A	0.0104	0.8448	0.1207	0.035*	
H24B	0.0389	0.8089	0.2622	0.035*	
H24C	0.1440	0.8577	0.2301	0.035*	

### Atomic displacement parameters (Å^2^)

**Table:**

	*U*^11^	*U*^22^	*U*^33^	*U*^12^	*U*^13^	*U*^23^
O1	0.0195 (4)	0.0190 (4)	0.0486 (6)	0.0025 (3)	0.0146 (4)	−0.0069 (4)
O2	0.0185 (4)	0.0128 (3)	0.0376 (5)	−0.0003 (3)	0.0030 (3)	−0.0021 (3)
O3	0.0148 (3)	0.0137 (3)	0.0323 (4)	0.0002 (3)	0.0084 (3)	0.0006 (3)
O4	0.0187 (4)	0.0191 (4)	0.0394 (5)	−0.0007 (3)	0.0131 (3)	0.0040 (3)
O5	0.0217 (4)	0.0293 (4)	0.0165 (4)	0.0057 (3)	0.0015 (3)	−0.0044 (3)
O6	0.0202 (4)	0.0194 (3)	0.0224 (4)	0.0060 (3)	0.0074 (3)	−0.0010 (3)
O7	0.0160 (3)	0.0176 (3)	0.0196 (4)	0.0032 (3)	0.0009 (3)	0.0009 (3)
O8	0.0217 (4)	0.0313 (4)	0.0257 (4)	0.0055 (3)	−0.0034 (3)	0.0020 (3)
C1	0.0160 (4)	0.0151 (4)	0.0225 (5)	0.0022 (3)	0.0059 (4)	0.0022 (3)
C2	0.0130 (4)	0.0153 (4)	0.0234 (5)	0.0004 (3)	0.0045 (4)	0.0005 (3)
C3	0.0156 (4)	0.0136 (4)	0.0283 (5)	0.0003 (3)	0.0039 (4)	−0.0018 (4)
C4	0.0172 (5)	0.0167 (4)	0.0326 (6)	0.0026 (3)	0.0078 (4)	−0.0031 (4)
C5	0.0171 (5)	0.0187 (5)	0.0485 (7)	−0.0010 (4)	0.0136 (5)	−0.0040 (5)
C6	0.0175 (5)	0.0156 (4)	0.0428 (7)	−0.0013 (4)	0.0106 (5)	−0.0032 (4)
C7	0.0147 (4)	0.0138 (4)	0.0263 (5)	0.0005 (3)	0.0057 (4)	−0.0005 (3)
C8	0.0151 (4)	0.0137 (4)	0.0220 (5)	0.0010 (3)	0.0030 (4)	−0.0002 (3)
C9	0.0154 (4)	0.0139 (4)	0.0184 (4)	0.0017 (3)	0.0041 (3)	0.0004 (3)
C10	0.0148 (4)	0.0137 (4)	0.0185 (4)	0.0010 (3)	0.0044 (3)	0.0007 (3)
C11	0.0156 (4)	0.0187 (4)	0.0176 (4)	0.0030 (3)	0.0022 (3)	0.0003 (3)
C12	0.0172 (4)	0.0179 (4)	0.0161 (4)	0.0009 (3)	0.0031 (3)	−0.0010 (3)
C13	0.0165 (4)	0.0153 (4)	0.0181 (4)	0.0029 (3)	0.0047 (3)	−0.0006 (3)
C14	0.0132 (4)	0.0143 (4)	0.0176 (4)	0.0009 (3)	0.0019 (3)	0.0012 (3)
C15	0.0184 (5)	0.0194 (4)	0.0194 (5)	−0.0014 (3)	0.0007 (4)	0.0014 (4)
C16	0.0205 (5)	0.0215 (5)	0.0167 (4)	−0.0011 (4)	0.0020 (4)	−0.0004 (4)
C17	0.0181 (4)	0.0156 (4)	0.0173 (4)	−0.0018 (3)	0.0049 (3)	−0.0005 (3)
C18	0.0152 (4)	0.0132 (4)	0.0165 (4)	0.0004 (3)	0.0043 (3)	0.0004 (3)
C19	0.0205 (5)	0.0279 (6)	0.0496 (8)	0.0011 (4)	0.0157 (5)	−0.0096 (5)
C20	0.0235 (6)	0.0236 (5)	0.0527 (8)	0.0009 (4)	0.0034 (5)	0.0119 (5)
C21	0.0184 (5)	0.0143 (4)	0.0451 (7)	−0.0001 (4)	0.0063 (5)	−0.0029 (4)
C22	0.0240 (5)	0.0293 (5)	0.0176 (5)	0.0024 (4)	−0.0006 (4)	−0.0004 (4)
C23	0.0184 (5)	0.0284 (5)	0.0322 (6)	0.0002 (4)	0.0078 (4)	−0.0034 (5)
C24	0.0285 (6)	0.0238 (5)	0.0178 (5)	0.0052 (4)	0.0057 (4)	−0.0028 (4)

### Geometric parameters (Å, °)

**Table:**

O1—C4	1.3548 (13)	C11—C12	1.3988 (14)
O1—C19	1.4386 (15)	C11—H11A	0.9300
O2—C3	1.3733 (12)	C12—C13	1.3939 (14)
O2—C20	1.4332 (16)	C13—C14	1.3911 (14)
O3—C2	1.3736 (12)	C14—C18	1.4107 (13)
O3—C1	1.3798 (12)	C15—C16	1.4421 (15)
O4—C1	1.2106 (13)	C16—C17	1.3569 (15)
O5—C12	1.3565 (13)	C16—H16A	0.9300
O5—C22	1.4305 (14)	C17—C18	1.4586 (14)
O6—C13	1.3723 (12)	C17—C24	1.5015 (15)
O6—C23	1.4419 (14)	C19—H19A	0.9600
O7—C14	1.3755 (12)	C19—H19B	0.9600
O7—C15	1.3777 (13)	C19—H19C	0.9600
O8—C15	1.2108 (13)	C20—H20A	0.9600
C1—C9	1.4594 (14)	C20—H20B	0.9600
C2—C3	1.3924 (14)	C20—H20C	0.9600
C2—C7	1.4023 (14)	C21—H21A	0.9600
C3—C4	1.4043 (15)	C21—H21B	0.9600
C4—C5	1.3980 (15)	C21—H21C	0.9600
C5—C6	1.3822 (15)	C22—H22A	0.9600
C5—H5A	0.9300	C22—H22B	0.9600
C6—C7	1.4016 (15)	C22—H22C	0.9600
C6—H6A	0.9300	C23—H23A	0.9600
C7—C8	1.4496 (14)	C23—H23B	0.9600
C8—C9	1.3605 (14)	C23—H23C	0.9600
C8—C21	1.4977 (14)	C24—H24A	0.9600
C9—C10	1.4945 (14)	C24—H24B	0.9600
C10—C11	1.3906 (14)	C24—H24C	0.9600
C10—C18	1.4168 (14)		
			
C4—O1—C19	116.67 (9)	O8—C15—O7	116.93 (10)
C3—O2—C20	112.74 (10)	O8—C15—C16	126.78 (10)
C2—O3—C1	121.26 (8)	O7—C15—C16	116.26 (9)
C12—O5—C22	117.03 (9)	C17—C16—C15	123.69 (10)
C13—O6—C23	112.73 (8)	C17—C16—H16A	118.2
C14—O7—C15	121.80 (8)	C15—C16—H16A	118.2
O4—C1—O3	116.55 (9)	C16—C17—C18	118.98 (9)
O4—C1—C9	125.29 (9)	C16—C17—C24	117.60 (10)
O3—C1—C9	118.15 (9)	C18—C17—C24	123.41 (9)
O3—C2—C3	116.39 (9)	C14—C18—C10	116.55 (9)
O3—C2—C7	121.30 (9)	C14—C18—C17	116.36 (9)
C3—C2—C7	122.30 (9)	C10—C18—C17	127.08 (9)
O2—C3—C2	120.39 (9)	O1—C19—H19A	109.5
O2—C3—C4	120.81 (9)	O1—C19—H19B	109.5
C2—C3—C4	118.76 (9)	H19A—C19—H19B	109.5
O1—C4—C5	124.40 (10)	O1—C19—H19C	109.5
O1—C4—C3	115.77 (10)	H19A—C19—H19C	109.5
C5—C4—C3	119.82 (10)	H19B—C19—H19C	109.5
C6—C5—C4	120.12 (10)	O2—C20—H20A	109.5
C6—C5—H5A	119.9	O2—C20—H20B	109.5
C4—C5—H5A	119.9	H20A—C20—H20B	109.5
C5—C6—C7	121.60 (10)	O2—C20—H20C	109.5
C5—C6—H6A	119.2	H20A—C20—H20C	109.5
C7—C6—H6A	119.2	H20B—C20—H20C	109.5
C6—C7—C2	117.31 (9)	C8—C21—H21A	109.5
C6—C7—C8	123.73 (9)	C8—C21—H21B	109.5
C2—C7—C8	118.93 (9)	H21A—C21—H21B	109.5
C9—C8—C7	118.78 (9)	C8—C21—H21C	109.5
C9—C8—C21	121.61 (9)	H21A—C21—H21C	109.5
C7—C8—C21	119.58 (9)	H21B—C21—H21C	109.5
C8—C9—C1	121.36 (9)	O5—C22—H22A	109.5
C8—C9—C10	122.93 (9)	O5—C22—H22B	109.5
C1—C9—C10	115.60 (9)	H22A—C22—H22B	109.5
C11—C10—C18	120.57 (9)	O5—C22—H22C	109.5
C11—C10—C9	115.52 (9)	H22A—C22—H22C	109.5
C18—C10—C9	123.91 (9)	H22B—C22—H22C	109.5
C10—C11—C12	121.19 (9)	O6—C23—H23A	109.5
C10—C11—H11A	119.4	O6—C23—H23B	109.5
C12—C11—H11A	119.4	H23A—C23—H23B	109.5
O5—C12—C13	115.33 (9)	O6—C23—H23C	109.5
O5—C12—C11	125.00 (9)	H23A—C23—H23C	109.5
C13—C12—C11	119.63 (9)	H23B—C23—H23C	109.5
O6—C13—C14	119.86 (9)	C17—C24—H24A	109.5
O6—C13—C12	121.34 (9)	C17—C24—H24B	109.5
C14—C13—C12	118.79 (9)	H24A—C24—H24B	109.5
O7—C14—C13	114.02 (8)	C17—C24—H24C	109.5
O7—C14—C18	122.74 (9)	H24A—C24—H24C	109.5
C13—C14—C18	123.23 (9)	H24B—C24—H24C	109.5
			
C2—O3—C1—O4	178.16 (10)	C8—C9—C10—C18	103.55 (13)
C2—O3—C1—C9	−0.94 (15)	C1—C9—C10—C18	−80.20 (12)
C1—O3—C2—C3	−175.74 (10)	C18—C10—C11—C12	1.57 (15)
C1—O3—C2—C7	4.15 (16)	C9—C10—C11—C12	−178.01 (9)
C20—O2—C3—C2	−103.96 (12)	C22—O5—C12—C13	−173.06 (9)
C20—O2—C3—C4	78.36 (14)	C22—O5—C12—C11	9.01 (16)
O3—C2—C3—O2	1.16 (16)	C10—C11—C12—O5	177.12 (10)
C7—C2—C3—O2	−178.72 (10)	C10—C11—C12—C13	−0.73 (16)
O3—C2—C3—C4	178.89 (10)	C23—O6—C13—C14	99.08 (11)
C7—C2—C3—C4	−1.00 (17)	C23—O6—C13—C12	−81.83 (12)
C19—O1—C4—C5	−7.65 (19)	O5—C12—C13—O6	2.19 (15)
C19—O1—C4—C3	172.97 (11)	C11—C12—C13—O6	−179.76 (9)
O2—C3—C4—O1	0.06 (17)	O5—C12—C13—C14	−178.71 (9)
C2—C3—C4—O1	−177.65 (10)	C11—C12—C13—C14	−0.66 (15)
O2—C3—C4—C5	−179.34 (11)	C15—O7—C14—C13	176.70 (9)
C2—C3—C4—C5	2.94 (18)	C15—O7—C14—C18	−4.07 (14)
O1—C4—C5—C6	178.29 (13)	O6—C13—C14—O7	−0.41 (14)
C3—C4—C5—C6	−2.4 (2)	C12—C13—C14—O7	−179.52 (9)
C4—C5—C6—C7	−0.2 (2)	O6—C13—C14—C18	−179.63 (9)
C5—C6—C7—C2	2.14 (19)	C12—C13—C14—C18	1.25 (15)
C5—C6—C7—C8	−175.98 (12)	C14—O7—C15—O8	−179.20 (9)
O3—C2—C7—C6	178.61 (11)	C14—O7—C15—C16	2.60 (14)
C3—C2—C7—C6	−1.51 (17)	O8—C15—C16—C17	−176.66 (11)
O3—C2—C7—C8	−3.18 (16)	O7—C15—C16—C17	1.34 (16)
C3—C2—C7—C8	176.71 (10)	C15—C16—C17—C18	−3.75 (16)
C6—C7—C8—C9	177.07 (11)	C15—C16—C17—C24	174.94 (10)
C2—C7—C8—C9	−1.03 (16)	O7—C14—C18—C10	−179.60 (9)
C6—C7—C8—C21	−0.87 (18)	C13—C14—C18—C10	−0.43 (14)
C2—C7—C8—C21	−178.97 (11)	O7—C14—C18—C17	1.53 (14)
C7—C8—C9—C1	4.21 (16)	C13—C14—C18—C17	−179.31 (9)
C21—C8—C9—C1	−177.89 (11)	C11—C10—C18—C14	−0.98 (14)
C7—C8—C9—C10	−179.74 (10)	C9—C10—C18—C14	178.57 (9)
C21—C8—C9—C10	−1.85 (17)	C11—C10—C18—C17	177.76 (10)
O4—C1—C9—C8	177.67 (11)	C9—C10—C18—C17	−2.69 (16)
O3—C1—C9—C8	−3.33 (15)	C16—C17—C18—C14	2.26 (14)
O4—C1—C9—C10	1.35 (16)	C24—C17—C18—C14	−176.35 (9)
O3—C1—C9—C10	−179.65 (9)	C16—C17—C18—C10	−176.47 (10)
C8—C9—C10—C11	−76.88 (13)	C24—C17—C18—C10	4.91 (16)
C1—C9—C10—C11	99.37 (11)		

### Hydrogen-bond geometry (Å, °)

**Table:**

*D*—H···*A*	*D*—H	H···*A*	*D*···*A*	*D*—H···*A*
C21—H21A···O6^i^	0.96	2.55	3.2921 (15)	134
C22—H22A···O6^ii^	0.96	2.52	3.4385 (14)	161
C22—H22B···O8^i^	0.96	2.56	3.4401 (15)	152
C6—H6A···Cg1^iii^	0.93	2.92	3.6706 (12)	138
C19—H19A···Cg2^iv^	0.96	2.60	3.5446 (14)	170

Symmetry codes: (i) −*x*, −*y*+2, −*z*+1; (ii) −*x*, −*y*+2, −*z*+2; (iii) *x*+1, *y*, *z*; (iv) *x*+1, −*y*+1/2, *z*−3/2.

## References

[bb1] Allen, F. H., Kennard, O., Watson, D. G., Brammer, L., Orpen, A. G. & Taylor, R. (1987). *J. Chem. Soc. Perkin Trans. 2*, pp. S1–19.

[bb2] Bernstein, J., Davis, R. E., Shimoni, L. & Chang, N.-L. (1995). *Angew. Chem. Int. Ed. Engl.* **34**, 1555–1573.

[bb3] Bruker (2005). *APEX2*, *SAINT* and *SADABS* Bruker AXS Inc., Madison, Wisconsin, USA.

[bb4] Carlton, B. D., Aubrun, J. C. & Simon, G. S. (1996). *Fundam. Appl. Toxicol.* **30**, 145–151. 10.1006/faat.1996.0051 8812259

[bb5] Cosier, J. & Glazer, A. M. (1986). *J. Appl. Cryst.* **19**, 105–107.

[bb6] El-Agrody, A. M., Abd El-Latif, M. S., El-Hady, N. A., Fakery, A. H. & Bedair, A. H. (2001). *Molecules*, **6**, 519–527.

[bb7] El-Farargy, A. F. (1991). *Egypt. J. Pharm. Sci.* **32**, 625–625.

[bb8] Emmanuel-Giota, A. A., Fylaktakidou, K. C., Hadjipavlou-Litina, D. J., Litinas, K. E. & Nicolaides, D. N. (2001). *J. Heterocycl. Chem.* **38**, 717–722.

[bb9] Ghate, M., Kusanur, R. A. & Kulkarni, M. V. (2005). *Eur. J. Med. Chem.* **40**, 882–887. 10.1016/j.ejmech.2005.03.025 16140424

[bb10] Kennedy, R. O. & Thornes, R. D. (1997). *Coumarins: Biology, Applications and Mode of Action* New York: Wiley & Sons.

[bb11] Laakso, J. A., Narske, E. D., Gloer, J. B., Wicklow, D. T. & Dowd, P. F. (1994). *J. Nat. Prod.* **57**, 128–133. 10.1021/np50103a018 8158157

[bb12] Nofal, Z. M., El-Zahar, M. & Abd El-Karim, S. (2000). *Molecules*, **5**, 99–113.

[bb13] Pratibha, S. & Shreeya, P. (1999). *Indian J. Chem. Sect. B*, **38**, 1139–1142.

[bb14] Shaker, R. M. (1996). *Pharmazie*, **51**, 148–148. 8900865

[bb15] Sheldrick, G. M. (2008). *Acta Cryst.* A**64**, 112–122. 10.1107/S0108767307043930 18156677

[bb16] Spek, A. L. (2009). *Acta Cryst.* D**65**, 148–155. 10.1107/S090744490804362X PMC2631630 19171970

[bb17] Yang, H., Protiva, P., Gil, R. R., Jiang, B., Baggett, S., Basile, M. J., Reynertson, K. A., Weinstein, I. B. & Kennelly, E. J. (2005). *Planta Med.* **71**, 852–60. 10.1055/s-2005-871257 16206041

[bb18] Zhou, P., Takaishi, Y. & Duan, H. (2000). *Phytochemistry*, **53**, 689–697. 10.1016/s0031-9422(99)00554-3 10746882

